# Antioxidant properties of *Ferulago angulata* and its hepatoprotective effect against N-nitrosodimethylamine-induced oxidative stress in rats

**DOI:** 10.1080/13880209.2016.1270974

**Published:** 2017-01-31

**Authors:** Hatice Kiziltas, Suat Ekin, Mahire Bayramoglu, Esvet Akbas, Gokhan Oto, Serkan Yildirim, Fevzi Ozgokce

**Affiliations:** a Medicinal and Aromatic Plants Program, Bitlis Eren University, Bitlis, Turkey;; b Department of Chemistry, Yuzuncu Yil University, Van, Turkey;; c Division of Biochemistry, Siirt University, Siirt, Turkey;; d Department of Pharmacology, Yuzuncu Yil University, Van, Turkey;; e Division of Patology, Ataturk University, Van, Turkey;; f Department of Biology, Yuzuncu Yil University, Van, Turkey

**Keywords:** *Ferulago angulata*, Antioxidant capacity, SOD, GSH-Px, CAT

## Abstract

**Context:***Ferulago angulata* (Schlecht.) Boiss. (Apiaceae) (FASB) is used to treat liver diseases and has been used both as food and therapeutics by many cultures for thousands of years because of the natural antioxidant compounds.

**Objective:** This study determines antioxidant properties of FASB flowers, the levels of minerals and vitamins, and also, evaluates the hepatoprotective effect of flowers against *N*-nitrosodimethylamine (NDMA) induced on liver tissue by assessing antioxidant enzymes and histopathological parameters in Wistar albino rats.

**Materials and methods:** In the study, the rats were divided into six groups of ten. Control, untreated animals were given 0.9% NaCl. Rats were intraperitoneally given NDMA (10 mg/kg) for the first 7 days. FASB methanol extract (150 and 300 mg/kg) was administered orally for 21 days.

**Results:** α-Tocopherol, retinol, ascorbic acid, total antioxidant activity, phenolic and flavonoid contents of FASB were 0.70 ± 0.13, 0.29 ± 0.03 μg/g, 139.32 ± 7.06 μg/100 g, 171.61 ± 6.05 mM ascorbic acid/g, 90.47 ± 4.11 mg GA/g and 37.39 ± 2.85 mg QE/g. DPPH and hydroxyl radical scavenging activity was obtained IC_50_ 67.34 ± 4.14 and 64.87 ± 4.68 μg/mL, respectively.

**Discussion and conclusion:** The results of the study indicated that FASB flowers contain high levels of vitamins, minerals, total antioxidant activity, phenolics and flavonoids. Due to the positive effect on significant changes in antioxidant enzymes of liver tissue and histopathological examination, it is thought that the plant could be used as a hepatoprotective.

## Introduction

*N*-Nitrosodimethylamine (NDMA) can be formed through a chemical reaction between monochloramine (DMA) and organic nitrogen compound (Kim & Clevenger 2007). It was put forth in previous studies that NDMA with strong carcinogenic properties can induce apoptosis in human leukocytes. The early and late phase responses to various pathogenic factors can be consequently impaired via increased induction of apoptosis and changes in the biological availability of the cells exposed to N-nitrosodimethylamine (Iwaniuk et al. 2015). Industries such as pesticide manufacturing, rubber, tanneries and alkylamine manufacture/use industries (foundries, fish processing), and dye manufacturing plants are places where occupational exposure to NDMA may take place (Ahmad et al. 2011).

Human sewage and industrial activities are known to be the primary sources of nitrosamines in water environments (Lee & Oh 2016). Formation of NDMA has been shown to occur during chloramine disinfection through a reaction between organic nitrogen containing precursor, such as dimethylamine and DMA (Andreas & David 2003). DMA or related compounds as well as ammonia and NDMA can be found in drinking waters and therefore should be considered as a potential disinfection by-products (Choi & Valentine 2002). The use of chlorine or chloramines as a primary disinfectant has been reported to increase NDMA concentrations in drinking water treatments (Kim & Clevenger 2007).

There are different ways with which drinking water can be exposed to NDMA. It can either result from direct ingestion as well as inhalation and dermal contact when showering, swimming in a chlorinated pool or bathing. Food is another source of exposure to NDMA in which secondary amines exposed to nitrate and/or nitrite during processing and preservation can form NDMA. Processed and/or preserved milk and milk products, meat and meat products, fish and fish products, baby foods, cereals, beverages, and vegetables and various fruits are among the common foods in which NDMA has been detected (Chowdhury 2014).

Hydroxyl radicals (OH^•^), superoxide anion (O_2_^−^) radicals, and hydrogen peroxide (H_2_O_2_) are among reactive oxygen species (ROS) which cause oxidative damage to macromolecules including DNA, proteins, lipids and small cellular molecules. The pathology of many diseases including atherosclerosis, cancer and diabetes have been implicated in free radicals (Fernández-Pachón et al. 2004; Esmaeili & Sonboli 2010; Wang et al. 2010; Kim et al. 2011; Nandhakumar & Indumathi 2013). It is known that many types of liver injuries are due to oxidative stress leading to the production of ROS. Free radical generation is stimulated by NDMA and ROS by hepatocyte. Liver fibrosis is induced by NDMA, a potent hepatocarcinogen and mutagen in addition to cirrhosis which is a consequence of repeated exposure of animals to its lower dosages (Areeba et al. 2011).

*Ferulago angulata* (Schlecht.) Boiss. (Apiaceae) (FASB) has been used to treat a wide range of activities such as aphrodisiac, helping digestion, for strengthening, as a sedative; it is good for relieve nervousness, headache, snakebite, haemorrhoid, ulcer and quite effective for especially digestive system diseases. The plant has been used in perfumery and cosmetic industries. It was reported that FASB has antioxidant and antidiabetic effects (Asghari et al. 2012), and may also have anticancer activity (Karimian et al. 2014).

A literature survey found no study reports the oxidative stress parameters of *Ferulago angulata* flower on rats or the oxidative stress studies formed with NDMA from any plant of the *Ferulago* genus. This study investigates the vitamin and mineral, total phenolic, and total flavonoid content, antioxidant capacity and the free radical scavenging activity of *Ferulago angulata* and its hepatoprotective effect on NDMA-induced liver toxicity was also investigated.

## Materials and methods

### Plant material

FASB flowers were collected in the Gurpinar district, Van, between Ozluce and Gecerli villages (July 2012, 38° 04′ 336″ N 43° 25′ 829″ E, 2010 m). FASB was identified by Dr Fevzi Ozgokce, Division of Botany. A specimen was deposited at the Department of Botany Herbarium, Yuzuncu Yil University, VANF F13982.

### Preparation of extracts

FASB flowers methanol extraction was performed according to the method of Cai et al. (2004) with slight modifications. FASB flowers were dried at room temperature. Samples were ground to a fine powder using a Kenwood Multi-Mill (Kenwood Ltd., UK) and passed through a sieve (24 mesh). In total, 5 g sample was extracted with 100 mL 80% methanol at 35 °C for 24 h. The samples were then cooled to room temperature, centrifuged at 5000 rpm for 10 min and filtered by Whatman No. 1 paper. Extracts were evaporated to dryness in a vacuum and lyophilized. Methanol extracts were placed in a dark bottle and stored at −20 °C until used.

### *In vitro* antioxidant activity studies

#### Total antioxidant activity

The method described by Prieto et al. (1999) was used to evaluate the total antioxidant activities in FASB methanol extracts. Reagent solution (28 mM sodium phosphate, 4 mM ammonium molybdate, and 0.6 M sulfuric acid) (2 mL) was mixed with 0.2 mL sample. A boiling water bath at 95 °C was used to incubate the tubes for 90 min. The samples were then cooled to room temperature, and the absorbance was measured at 695 nm against the blank. The number of ascorbic acid equivalents in mM per gram extract is used to the total antioxidant activity.

#### DPPH radical scavenging activity

DPPH methanol solution (5 mL; 0.004%) was mixed with 50 μL extract at various methanol concentrations. The absorbance was measured against a blank at 517 nm following an incubation period at room temperature of a 30 min (Chen et al. 2009; Cuendet et al. 1997). IC_50_ values were used to express sample antioxidant activity which was calculated from inhibition plot. The synthetic antioxidant butylated hydroxytoluene (BHT) was used as a standard control in the experiments.

#### Superoxide anion radical scavenging activity

The method of Zhishen et al. (1999) was used to generate superoxide radicals. Riboflavin-light-NBT system lays the foundation of the assay for O_2_^−^ radical scavenging activity. Riboflavin, methionine, and illuminate were used to generate superoxide radicals which were then assayed by the reduction of NBT to form blue formazan. All solutions were prepared in 0.05 M phosphate buffer (pH 7.8). A fluorescent lamp which illuminates the reaction mixtures containing the different concentrations of FASB (10–120 μg/mL) methanol extracts were used to initiate the reaction. The total volume of the reaction mixture (3 mL) was illuminated at 25 °C for 40 min. The absorbance was measured at 560 nm. BHT and α-tocopherol were used as positive control.

#### Scavenging of hydroxyl radical

The method modified by Kunchandy and Rao (1990) was used to measure the OH^•^ scavenging of FASB methanol extracts. Ferric chloride (1 mM), 3 mM deoxyribose in 20 mM phosphate buffer pH 7.4, 1 mM EDTA solution, and 20 mM H_2_O_2_ with or without each extract solution formed the reactive mixture. The reaction was incubated for 60 min at 37 °C. A solution of 1% TBA in 1 mL 2.8% TCA was added to the mixture which was then incubated for 30 min in a boiling water bath. The absorbance was recorded as 532 nm following cooling.

#### Hydrogen peroxide scavenging activity

The scavenging activity for H_2_O_2_ was measured according to method of Ruch et al. (1989). Different concentrations of plant extract were added to 2 mL of H_2_O_2_ solution (10 mM) in phosphate buffer (pH 7.4, 50 mM) and there action mixture was incubated at 25 °C for 30 min. The unreacted H_2_O_2_ was determined by measuring the absorbance of their action mixture at 230 nm with respect to the blank solution.

#### Total phenolic

Modified Folin Ciocalteu method of Singleton et al. (1999) was used to measure the total phenolic content in FASB methanol extracts. Methods involving gallic acid as standard and Folin–Ciocalteu reagent were employed to determine the total soluble phenolic constituents of the FASB flower methanol extracts. Extract solution (0.1 mL) containing 1 mg extract was obtained in a calibrated flask after which 1 mL Folin–Ciocalteu reagent and 45 mL distilled water were added and the flask was thoroughly shaken. Na_2_CO_3_ solution (3 mL) (2%, w/v) was added after 3 min after which the mixture was allowed to stand for 2 h with intermittent shaking. Absorbance was measured at 760 nm (Yi et al. 1997; Gamez-Meza et al. 1999). Gallic acid equivalents per gram (mg GA/g) extract is used to express phenolic content.

#### Total flavonoids

Aluminum chloride colorimetric method with quercetin as a standard was used to determine the total ﬂavonoid content the results of which were expressed in mg quercetin equivalents. In short, 1 mL 2% AlCl_3_ in methanol was mixed with the same volume of methanol extracts (2 mg). Absorption readings at 394 nm were taken after 10 min against a blank sample consisting of 1 mL extract solution with 1 mL methanol (Lamasion et al. 1990; Urgeova & Polivka 2009).

#### Determination of metal content

Trace elements (Fe, Zn, Cu, Mn, Cr, Co), minerals (Mg, Ca, K, Na) analysis ICP-OES (Thermo iCAP 6300 DUO, England) were used in this study. All metal concentrations were determined on a dry weight basis. Multi-element reference materials (Inorganic ventures IV-Stock-8) were used.

### Vitamin (A, E, C) analysis

#### Standard solutions and calibration

**Figure 1.**
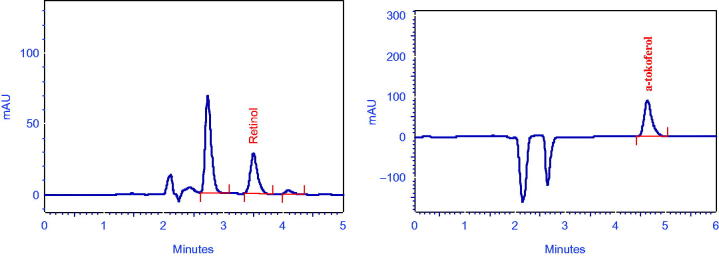
Isocratic elution was performed with methanol-THF (80:20, v/v) as mobile phase at a flow rate of 1.5 mL/min, 40 °C. C_18_ reversed phase column (250 × 4.6 mm ID) was employed. The chromatogram was monitored with photodiode array detector (PDA) array detection at 325 and 290 nm α-tocopherol and all-trans-retinol.

α-Tocopherol and all-trans-retinol stock solutions were prepared at 500 μg/mL in methanol (Figure 1). Vitamin C stock solution was prepared at 4000 mg/mL in metaphosphoric acid. Stock solutions were appropriately diluted with the methanol for standard solution preparation. Calibration was calculated by linear regression analysis of the peak area versus the standard solution concentrations.

#### Extraction process

FASB (5 g) was extracted by hexane and ethyl alcohol (containing 0.01% BHT), and the sample was vortexed for 1 min (Su et al. 2002). The sample was extracted for 30 h in darkness. The sample was vortexed and centrifuged at 8000 rpm for 12 min. The supernatant was filtered using Whatman filter paper, and 500 μL of the hexane layer was extracted and evaporated to dryness under a nitrogen stream at 37 °C. The residue was dissolved in tetrahydrofuran (50 μL) and methanol was added (150 μL). The sample was vortexed for 1 min then 100 μL samples were autosampled using amber glass vials.

#### Chromatographic conditions

The chromatographic system consisted of Thermo Scientific Finnigan Surveyor with a photodiode Array (PDA) detector and a tray autosampler (−8 °C). The data were processed with Thermo Scientific ChromQuest version 4.2 software (Thermo Fisher Scientific, Waltham, MA). Separation was performed with a 5 μm Gl Science C_18_ reverse phase column (250 × 4.6 mm ID). The mobile phase of a methanol-THF mixture (80:20, v/v) was modified from Bruni et al. (2004), Saleh et al. (2006) and Sahin et al. (2005). Pump was set at a flow rate of 1.5 mL/min. The chromatographic analysis was performed at 40 °C with isocratic elution. The chromatogram was monitored with photodiode array detector (PDA) array detection at 325 and 290 nm (α-tocopherol and all-trans-retinol, respectively).

#### Vitamin C analysis

Vitamin C stock solution was prepared at 4000 mg/mL in metaphosphoric acid. Stock solutions were appropriately diluted with double distilled water for standard solution preparation. Vitamin C determination was performed from the homogenized dried FASB samples. The vitamin C method used was modified according to Omaye et al. (1979), Golubkina & Prudnik (1989) and Brewster (1984). Vitamin C content of FASB samples were measured spectrophotometrically (Shimadzu UV 1800, Japan) at 521 nm according to 2,4-dinitrophenyl hydrazine (DNPH) method.

#### Experimental animals

In total, 60 male Wistar albino rats (200 ± 50 g weight; 4 weeks old) were used in the study. Rats were obtained from the Animal Care Center, School of Medicine, Yuzuncu Yil University. Wistar albino rats were housed in a temperature-controlled room (22 ± 2 °C) with a 12 h light/dark cycle; food and water were given *ad libitum*. Experiments were performed according to the guidelines of the animal ethics committee of the Institute. The study protocol was approved by the Yuzuncu Yil University Animal Experiments Ethic Committee (YUHADYEK, 28.03.2013/Decision no: 2013/03).

#### Experimental design

The experimental period was performed with 60 male Wistar albino rats. The rats were randomly divided into six groups of ten rats each. Group I: Control, untreated animals were given normal saline (0.9% NaCl), Group II: The NDMA-treated animals were administered NDMA (10 mg per kg body weight) via intraperitoneal injection in the first seven days of the study. Group III: It was determined as FASB group. FASB (150 mg per kg body weight) were daily administered to the rats by an intra-gastric tube, Group IV: It was determined as NDMA + FASB group. FASB flowers extract (150 mg/kg) were administered to the rats in group orally daily and 10 mg/kg NDMA were administered in the first 7 days of study. Group V: It was determined as FASB group. FASB flowers extract (300 mg/kg) were administered to the rats in group each day. Group VI: It was determined as NDMA + FASB. FASB flowers extract (300 mg/kg) for each day and 10 mg/kg NDMA in the first 7 days of study were administered to the rats in group. The experimental period was continued for 21 days. At the end of the experimental period, all the rats were sacrificed; livers were removed and immediately processed for histopathological studies.

#### Plant material and extract preparation

Dried FASB flower (3000 mg) was infused in 300 mL boiled distilled water for 15 min. After filtration, the filtrate was again dried in an incubator at a 37 °C. The aqueous extracts were then obtained daily in isotonic physiological solution (0.9% NaCl). Extract was used to assess hepatoprotective properties.

### Antioxidant enzymes assay

#### Liver tissue preparation

The rats were anesthetized by diethyl ether inhalation and sacrificed. Liver tissues were washed with 0.9% NaCl, tissues were stored at −80 °C until analysis. Tissue samples (0.5 g) were homogenized in 5 mL ice-cold homogenization buffer (1 mmol/L EDTA, 0.32 mol/L sucrose and 10 nmol/L Tris-HCl, pH 7.4), using a homogenizer (Ultra Turrax T25, IKA, Staufen, Germany) and a glass-porcelain homogenizer (20 KHz frequency ultrasonic, Bandelin Sonupuls) for 8 min and then centrifuged at 9500 rpm for 30 min (Xia et al. 1994). All of the processes were performed at +4 °C, and then the clear upper supernatants were removed for analyses. Supernatants were used to determine antioxidant enzyme activity.

#### Antioxidant enzymes estimation

The clear supernatant obtained from the liver tissue homogenate was used to assess endogenous antioxidant enzymes superoxide dismutase (SOD), glutathione peroxidase (GSH-Px) and catalase (CAT). While tissue SOD (EC 1.15.1.1) activity was determined at 505 nm wavelength according to method of Sun et al. (1988), GSH-Px (E.C.1.11.1.9) tissue enzyme activity was measured at 340 nm wavelength by the method of Paglia and Valentine (1967), CAT (EC 1.11.1.6) activity was estimated at 240 nm by the method described by Aebi (1984). The results were expressed as IU/mg protein (wet tissue weight) in terms of specific activity.

#### Total protein concentration determination

Total protein concentration of liver homogenates was determined employing the method of Lowry et al. (1951) using bovine serum albumin as a standard. Different concentrations (10–200 μg) of standard protein bovine serum albumin were processed for preparation of a standard curve. Measurements were performed against blank at 695 nm wavelength. The values were expressed as mg protein/g wet tissue (mg/g).

#### Histopathological analysis of the liver

At the end of the treatments, the rats were sacrificed by decapitation. The livers were removed, washed immediately with saline, fixed in 10% formalin solution for 24 h and embedded in paraffin. After preparing paraffin blocks, consecutive 5 μm thick sections of the livers were cut using a microtome and stained with haematoxylin and eosin and Masson’s trichrome. The sections were examined under light microscopy (Nikon, Tokyo, Japan) for histopathological changes.

### Statistical analysis

Results are expressed as X ± SEM analysis of variance (ANOVA) was performed, and the statistical comparisons among the groups were carried out with *post hoc* Tukey's test for normally distributed variables, or with nonparametric Benferroni test for non-normally distributed data using a statistical package program (SPSS 22.0 for Windows). Nonlinear regression analysis was used to calculate the IC_50_ values.

## Results

Table 1 demonstrates α-tocopherol, retinol, vitamin C, total antioxidant activity, phenolic and ﬂavonoid, trace element (Fe, Zn, Cu, Mn, Cr, Co) and mineral (Mg, Ca, K, Na) content of methanol extracts of FESB flowers.

**Table 1.** Vitamin (E, A, C), total antioxidant activity, total phenolic and flavonoid, trace element (Fe, Zn, Cu, Mn, Cr, Co) and mineral (Mg, Ca, K, Na) content in *FESB* flower samples.

	*FESB*
Parameters	X ± SEM
α-Tocopherol (μg/g)	0.70 ± 0.13
Retinol (μg/g)	0.29 ± 0.03
Vitamin C (μg/100 g)	139.32 ± 7.06
Total phenolic content (mg GA/g)	90.47 ± 4.11
Total flavonoid content (mg QE/g)	37.39 ± 2.85
T. antioxidant capacity (mM AA/g)	171.61 ± 6.05
Fe (μg/g)	33.69 ± 0.18
Zn (μg/g)	16.97 ± 1.11
Cu (μg/g)	0.83 ± 0.04
Mn (μg/g)	7.51 ± 0.08
Cr (μg/g)	0.29 ± 0.03
Co (μg/g)	0.037 ± 0.002
Mg (μg/g)	299.0 ± 1.89
Ca (μg/g)	895.4 ± 2.73
K (μg/g)	4633 ± 17.12
Na (μg/g)	47.94 ± 1.06

### Vitamins E, A, C and trace elements

Figure 1 shows at 325 and 290 nm α-tocopherol and all-*trans*-retinol chromatograms (Figure 1). Vitamin A concentrations in FESB flowers was 0.29 ± 0.03 μg/g, vitamin E content in the flowers was 0.70 ± 0.13 μg/g. Vitamin C content was 139.32 ± 7.06 μg/100 g on dry weight basis. While evaluating the results, it was observed that FESB flowers contained vitamins A, E, and C in significant levels.

The contents of iron, zinc, copper, manganese, chromium, cobalt, magnesium, calcium, potassium, and sodium in of FESB flowers were found 33.69 ± 0.18, 16.97 ± 1.11, 0.83 ± 0.04, 7.51 ± 0.08, 0.29 ± 0.03, 0.037 ± 0.002, 299.0 ± 1.89, 895.4 ± 2.73, 4633 ± 17.12 and 47.94 ± 1.06 μg/g, respectively, on dry weight basis.

### *In vitro* antioxidant properties studies

#### Total phenolic and flavonoid

Total phenolic and ﬂavonoid content in the present study was evaluated in methanol extracts of FESB. Phenols are commonly found in plants and reportedly have several biological activities including antioxidant activities. Total phenolic content of FESB methanol extracts was 90.47 ± 4.11 mg GA/g. Flavonoid contents of the FESB methanol extracts were 37.39 ± 2.85 mg QE/g.

#### Total antioxidant activity

The present study was performed to evaluate total antioxidant activity of the FESB flowers methanol extracts. Total antioxidant activity of methanol extracts from the FESB was 171.61 ± 6.05 mM ascorbic acid/g. FESB flowers extracts exhibited effective and powerful antioxidant activity.

#### DPPH, hydroxyl, superoxide radical and hydrogen peroxide scavenging property

The DPPH, OH^•^, O_2_^−^ radical and H_2_O**_2_**scavenging activities of FESB flowers methanol extracts along with the standard references BHT and α-tocopherol are shown in Table 2.

**Table 2.** Percent values of DPPH, OH^•^, O_2_^−^, H_2_O_2_ and Concentrations (IC_50_), which inhibit DPPH^•^ and OH^•^ radicals at the rate of 50% in methanol extracts of *FESB* flowers compared with a positive controls.

	DPPH^•^		OH^•^		O_2_^-^	H_2_O_2_
	% Inhib. X plusmn; SEM	IC_50_ (μg/mL) X ± SEM	% Inhib. X ± SEM	IC_50_ (μg/mL) X ± SEM	% SARSA X ± SEM	% HPSA X ± SEM
*FESB*	70.82 ± 0.76	67.34 ± 4.14	68.67 ± 1.39	64.87 ± 4.68	51.58 ± 5.65	34.37 ± 12.28
BHT	51.95 ± 6.04	137.1 ± 0.59	66.04 ± 0.89	89.91 ± 4.13	47.96 ± 2.83	32.00 ± 2.16
α-toc.	–	–	–	–	36.20 ± 9.07	24.51 ± 4.04

**Figure 2.**
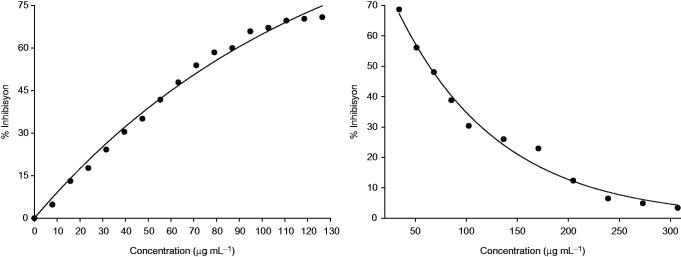
Inhibition of DPPH and OH&cenveo_unknown_entity_wingdings_F09F; radicals versus concentrations of *FESB* flower methanol extract.

In our study, we determined the DPPH radical scavenging activity of the FESB flowers methanol extracts, and we compared it with BHT, a synthetic antioxidant known to have antioxidant features. The highest radical inhibition rate of the FESB extract was 70.82 ± 0.76 and the BHT rate was 51.95 ± 6.04%. The sample concentration that was necessary to reduce the initial DPPH concentration by 50% (IC_50_) under the experimental conditions was determined. The best free radical scavenging activity was obtained with the FESB methanol extract (IC_50_ 67.34 ± 4.14 μg/mL) while BHT demonstrated comparable free radical scavenging activity with an IC_50_ value of 137.1 ± 0.59 μg/mL (Figure 2). It was observed that the FESB flowers methanol extract was more active in scavenging DPPH radicals compared to the positive control BHT.

The hydroxyl radical scavenging activity was obtained with the methanol extract (IC_50_ 64.87 ± 4.68 μg/mL) comparable levels of BHT with an IC_50_ value of 89.91 ± 4.13 μg/mL. Thus, the FESB methanol extract demonstrated more potent *in vitro* antioxidant activity with higher inhibition, than the BHT. The highest hydroxyl radical inhibition rates were 68.67 ± 1.39% for the FESB extracts and 66.04 ± 0.89% for BHT. Standard antioxidant substance (BHT) was more effective in scavenging radicals than FESB extract (Figure 2).

It was observed that the FESB methanol extracts were highly effective in scavenging superoxide radicals. The highest radical-inhibiting rates were 51.58 ± 5.65% for FESB 47.96 ± 2.83% for the BHT and 36.20 ± 9.07 for α-tocopherol. FESB methanol extract have a high radical scavenging effect, demonstrating superoxide radical scavenging activity in lower concentrations (40 μg/mL).

Regarding H_2_O**_2_** scavenging activity (%), it was found that FESB methanol levels were comparable with BHT and α-tocopherol. The highest radical-inhibiting rates were 34.37 ± 12.28% for FESB 32.00 ± 2.16% for the BHT and 24.51 ± 4.04 for α-tocopherol in (150 μg/mL). The results showed that the FESB methanol extract had antioxidative potential, similar to positive control (BHT).

Our study found that FESB is rich in vitamins, mineral, total phenol and total flavonoid content; therefore, its antioxidant capacity is quite high. Moreover, it is quite effective in scavenging stable DPPH free radicals and OH^•^, O_2_^−^ radicals; thus, it has anti-radical activity. The antioxidant and other functional properties may be due to the presence of phenolic compounds.

#### Antioxidant enzymes assay

Enzymatic antioxidant activities (SOD, CAT and GSH-Px) were studied in controls and different groups that were subjected to *in vivo* NDMA-induced oxidative stress. Levels of antioxidant enzyme SOD, CAT and GSH-Px activities are presented in Table 3.

**Table 3.** Antioxidant enzymes (SOD, GSH-Px and CAT) activities in control, NDMA, *FASB* (150 mg/kg), NDMA + *FASB* (150 mg/kg), *FASB* (300 mg/kg) and NDMA + *FASB* (300 mg/kg) groups in liver tissue samples.

Groups	SOD (IU/mg prot.) X ± SEM	GSH-Px (IU/mg prot.) X ± SEM	CAT (IU/mg prot.) X ± SEM
Control	45.13 ± 3.36^b^	1.64 ± 0.14^a,c^	2.15 ± 0.21^c,a,b,a1^
NDMA	31.77 ± 1.24^b,c^	0.77 ± 0.06^a,a1,a2,c1^	0.79 ± 0.07^a1,a2,a3^
FASB^*^	40.57 ± 3.83	1.44 ± 0.13^a1^	1.60 ± 0.13^c,c1,a2^
NDMA + FASB*	34.68 ± 1.14	1.16 ± 0.05^c^	1.01 ± 0.06^a,c1,b1^
*FASB*#	41.54 ± 2.22^c^	1.48 ± 0.08^a2^	1.79 ± 0.09^b1,a3^
NDMA+ *FASB*#	38.05 ± 3.41	1.27 ± 0.11^c1^	1.36 ± 0.18^b^

^a,a1,a2,a3^*p* < 0.001, ^b,b1^*p* < 0.01, ^c,c1^*p* < 0.05 (different letters, significant differences between groups). FASB* (150 mg/kg), *FASB*# (300 mg/kg).

**Figure 3.**
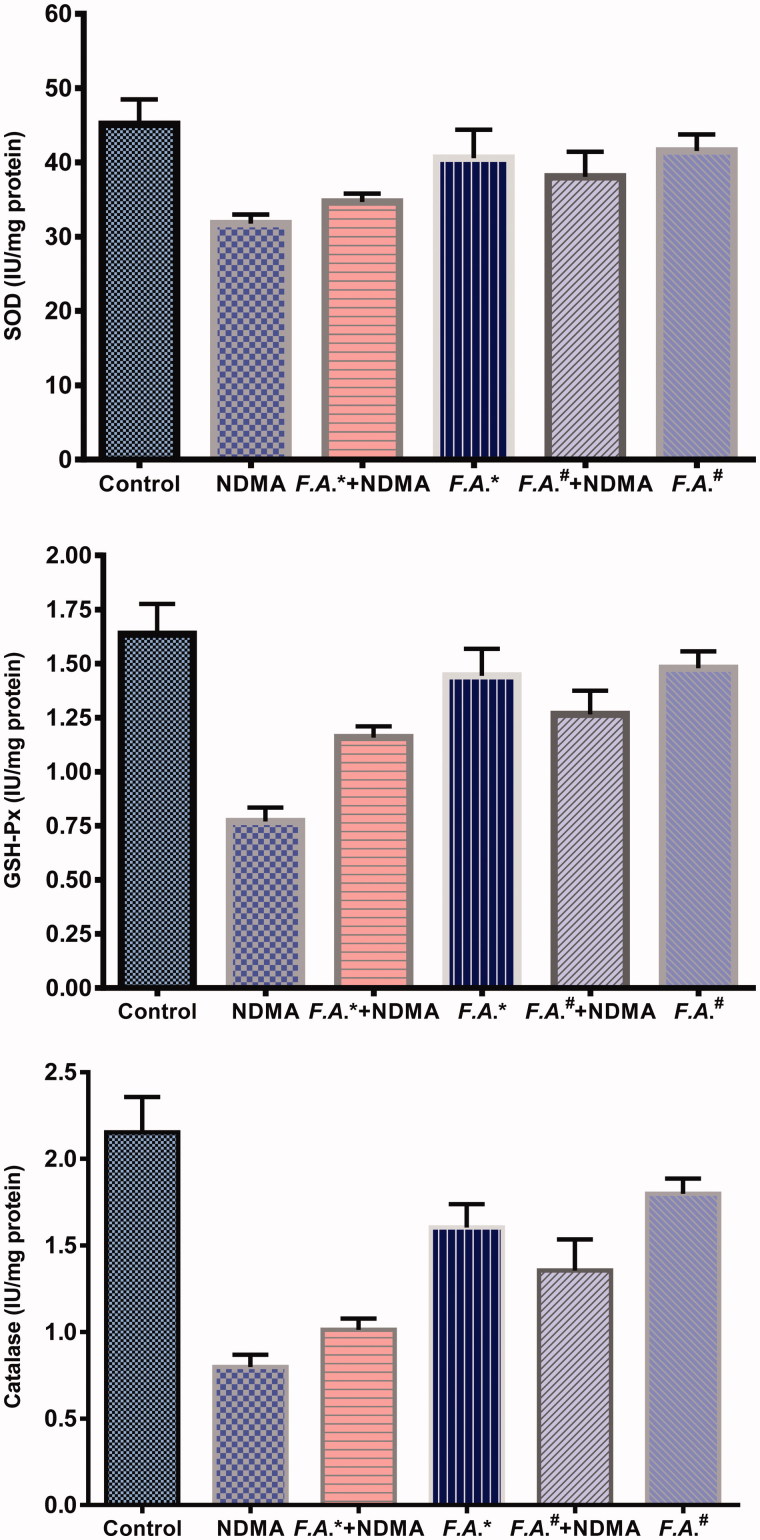
Antioxidant enzyme (SOD, GSH-Px and CAT) activities in liver tissue samples of control, NDMA, NDMA +150 mg/kg *FESB*^(*)^,150 mg/kg *FESB*^(*)^,NDMA +300 mg/kg *FESB*# and 300 mg/kg *FESB*# groups.

Antioxidant enzyme activities which were determined in rat liver tissue, following statistical analysis in GSH-Px, SOD, CAT enzyme activities, NDMA group was significantly lower than control group, (*p* < 0.001, *p* < 0.01, *p* < 0.001), respectively. Between control with FASB (150 mg/kg) group, CAT enzyme activity significantly (*p* < 0.05) decreased. Control and NDMA + FASB (150 mg/kg) groups enzyme activities of GSH-Px and CAT significantly decreased (*p* < 0.05 and *p* < 0.001). Between control with NDMA + FASB (300 mg/kg) groups, CAT enzyme activity significantly (*p* < 0.01) decreased. Whereas, NDMA group with FASB (300 mg/kg) group in the SOD, GSH-Px and CAT enzyme activities (*p* < 0.05, *p* < 0.001, *p* < 0.001), respectively, with FASB (150 mg/kg) groups in the GSH-Px and CAT enzyme activities (*p* < 0.001 and *p* < 0.001), and with NDMA + FASB (300 mg/kg) group in the GSH-Px enzyme activity (*p* < 0.05), statistically significantly increase was determined. However, in the CAT activity significant decrease was observed in NDMA + FAS*B* (150 mg/kg) treatment group as compared to FASB (150 mg/kg) and FASB (300 mg/kg) groups (*p* < 0.05 and *p* < 0.01) (Figure 3).

#### Histopathological analysis of liver

**Figure 4.**
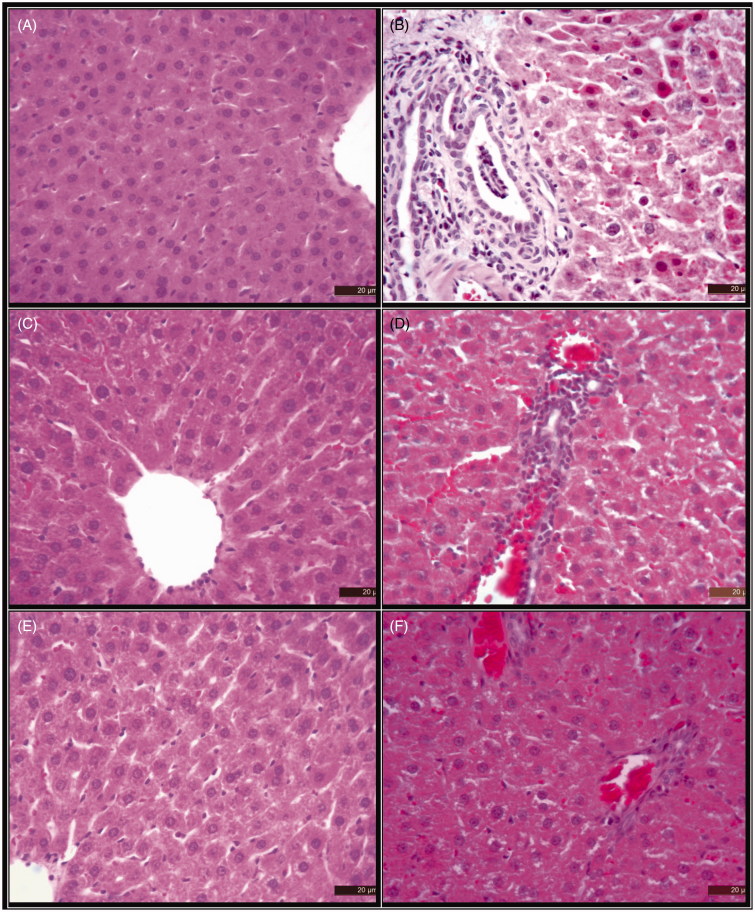
Histochemical localization by H & E staining of the liver of (A) control (0.9% NaCl), (B) NDMA treated, (C) *FESB* (150 mg**/**kg), (D) NDMA+ *FESB* (150 mg**/**kg treated), (E) *FESB* (300 mg**/**kg treated) and (F) NDMA+ *FESB* (300 mg**/**kg) treated experimental animals. Biopsies of liver from Group A showing normal structure Bar: 20 μ. Section of the liver from Group B rats reveals liver hyperaemic, sinusoidal dilatation, perivascular cell infiltration, fibrosis in the portal area, degeneration and necrosis in the hepatocytes Bar: 20 μ. Section of liver from Group C, normal histologic structure. Section of liver from Group D, a small number of perivascular cell infiltration, hyperaemia, degeneration in the hepatocytes. Section of liver from Group E, rats showing normal histologic structure. Section of liver from Group F, decreased degree of degeneration in the hepatocytes and liver hyperemic Bar: 20 μ.

Liver tissues were stained with haematoxylin and eosin and visualized under light microscopy. Figure 4 illustrated a light micrograph of a histopathological examination of the liver of control, NDMA-treated, FASB (150 mg/kg). NDMA + FASB (150 mg/kg), FASB (300 mg/kg) and NDMA + FASB (300 mg/kg) treated experimental animals.

The degree of hepatic damage due to NDMA treatment was examined by hematoxylin and eosin (H&E) staining of liver sections of rats. A section of liver from the control group (0.9% NaCl) showed normal lobular structure. A section of the liver from NDMA group reveals liver hyperaemic, sinusoidal dilatation, perivascular cell infiltration, fibrosis in the portal area, degeneration, and necrosis in the hepatocytes. Section of liver from FASB (150 mg/kg) group normal histologic structure. Section of liver from NDMA + FASB (150 mg/kg) group a small number of perivascular cell infiltration, hyperaemia, and degeneration in the hepatocytes. Section of liver from FASB (300 mg/kg) group, rats showed typical lobular structure. Section of liver from NDMA + FASB (300 mg/kg) group, decreased degree of degeneration in the hepatocytes and liver hyperaemic.

## Discussion

The present study was performed to investigate vitamin (A, E, C), trace element (Fe, Zn, Cu, Mn, Cr, Co) and mineral (Mg, Ca, K, Na) content as well as the *in vitro* antioxidant potential (total antioxidant activity, DPPH, hydroxyl radical, superoxide anion, H_2_O**_2_** scavenging activity, total phenolic and flavonoid content) of a methanol extract of the FESB flowers, as well as possible *in vivo* hepatoprotective properties against NDMA-induced oxidative stress in rats.

Vitamins A, E, and C are among the main antioxidant vitamins. They play an important role in the protection of cell components from oxidative damage (McDowell et al. 2007). No study was found on the levels of vitamin A, E, and C in the FASB flower. However, level of vitamin C in *Petroselinum crispum* (Apiaceae), a significant species, was determined as 133.0 mg/100 g in a study performed by Caunii et al. (2010). Level of vitamin E in *Petroselinum crispum* was reported as 0.75 mg/100 g whereas level of vitamin A in *Petroselinum crispum* was put forth as 421 μg/100 g (USDA 2015). FASB is a good source of vitamin E (0.70 ± 0.13 μg/g) and vitamin C (139.32 ± 7.06 μg/100 g) comparable with *Petroselinum crispum*.

No study was determined on the trace metal level of FASB flower. However, *Petroselinum crispum* had levels of mineral were 81.26 ± 7.00 mg/kg Fe, 19.32 ± 0.35 mg/kg Zn, 4.21 ± 0.13 mg/kg Cu, 50.00 mg/100 g Mg, 138.00 mg/100 g Ca, 554 mg/100 g K, 56 mg/100 g Na (Dghaim et al. 2015; USDA 2015). When FASB flower was compared with the *Petroselinum crispum*, it was determined that the levels of Mg (299.0 ± 1.89 μg/g, Ca (895.4 ± 2.73 μg/g), and K (4633 ± 17.12 μg/g) are higher and that potassium is especially quite high.

It was determined that methanol extract of FASB is more effective in comparison to BHT which is used as a positive control in DPPH radical scavenging and FASB is effective in DPPH radical scavenging. It was determined that methanol extract of the plant is more effective in comparison with BHT which is used as a positive control in hydroxyl radical scavenging and that FASB is effective in OH^•^ radical scavenging. Superoxide anion radical scavenging activity found in 40 μg/mL concentration of standard antioxidant substances (BHT and α-tocopherol), which was used in study with methanol extract of FASB flower in our study. In 40 μg/mL concentration, when results were compared with standards, it was determined that FASB flowers effectively scavenged superoxide anion radical in comparison with BHT and α-tocopherol standards.

Hydrogen peroxide scavenging activity in 150 μg/mL concentration was determined When results were compared with standards, it was determined that the FASB flower is more effective in H_2_O**_2_** radical scavenging as against α-tocopherol, also, antioxidative potential of FESB, similar to positive control (BHT).

Antioxidant enzyme activities which were determined in rat liver tissue, following statistical analysis in GSH-Px, SOD, CAT enzyme activities. Statistical analysis showed that the NDMA group was significantly lower than the control group with regards to GSH-Px, SOD, CAT enzyme activities (*p* < 0.001, *p* < 0.01, *p* < 0.001, respectively). On the other hand the FASB (150 mg/kg), NDMA + FASB (150 mg/kg) and NDMA + FASB (300 mg/kg) groups were also significantly lower than the control group regarding CAT enzyme activity (*p* < 0.05), regarding GSH-Px and CAT enzyme activities (*p* < 0.05, *p* < 0.001, respectively) and regarding CAT enzyme activity (*p* < 0.01), respectively. Whereas the NDMA group had increased enzyme activities of SOD, GSH-Px and CAT according to FASB (300 mg/kg) group (*p* < 0.05, *p* < 0.001, *p* < 0.001, respectively), regarding GSH-Px and CAT enzyme activities with FASB (150 mg/kg) (*p* < 0.001, *p* < 0.001, respectively) and enzyme activity of GSH-Px according to NDMA + FASB (300 mg/kg) group (*p* < 0.05). Similarly, NDMA + FASB (150 mg/kg) group had significantly lower enzyme activity of CAT than FASB (150 mg/kg) and FASB (300 mg/kg) groups (*p* < 0.05, *p* < 0.01, respectively).

Limited studies have been carried out with regard to studies on damage caused by NDMA and effects of NDMA on antioxidant defensive system, in which different plant species and effects of antioxidant substances have been examined. In a study carried out by Ahmad and Ahmad (2014) fibrosis was formed using NDMA on rat liver and they examined the effects of resveratrol on liver damages. It was determined as a result of the evaluations carried out that oxidative damages formed with NDMA in rats caused reduction in liver tissue SOD enzyme activity at a rate of 65% (*p* < 0.01). It was observed that levels of SOD specific enzyme activity increased in the group to which resveratrol was administered together with NDMA.

Hepatotoxicity was formed via NDMA on Swiss-Albino rats and protective effects of different doses of *Operculina turpethum* (Convolvulacaceae) were evaluated in another study (Sharma & Singh 2014). As a result this, which lasted 21 days, the enzyme activity levels of SOD, CAT, GSH, AST, ALT, ALP enzymes had significant antioxidant activity on rat liver tissues; they also examined liver tissue samples histopathologically. The rat liver SOD enzyme activities in the control and NDMA groups were determined as 9.112 ± 0.004 U/mL and 2.501 ± 0.027 U/mL, respectively, and it was quite apparent that there was a reduction in enzyme activity levels. They determined that *O. turpethum* extracts at different doses which, when applied to the groups, caused an increase in CAT and SOD enzyme activity in comparison with the NDMA group. In the consequence of histopathologic evaluations performed on liver tissues of groups, they determined that normal hepatocyte cells, normal lobular structure and central vein were seen in control group and that there were perivenular necrosis and significant expansion in veins and beginning of dense necrosis, fibrosis and sinusoidal dilatation in NDMA administrated group.

As a result of *in vivo* studies, it was observed that FASB flower may have a positive effect on liver tissues of rats with inflicted damage due to NDMA which is a substance that has negative effects on liver functions. It was determined that in NDMA group, as a result of damage that occurred, GSH-Px, SOD and CAT specific enzyme activity levels of liver tissues statistically reduced as against control group (Figure 3). Decreased enzyme activities (GSH-Px, SOD, CAT) in NDMA-induced oxidative stress may be due to the generation of increased ROS such as OH^•^, O_2_^−^ and DPPH radical scavenging activity. In addition, it was also observed that SOD, CAT and GSH-Px enzyme activity levels that reduced as a consequence of the damage increased statistically in comparison with the groups with NDMA damage in liver tissues of those groups that FASB flower was applied together with NDMA. It was supposed that FASB flower has an important effect in suppressing oxidative stress caused by NDMA and that overdoses of flower may be more effective in preventing damage occurring on antioxidant enzymes and liver tissues. The results were confirmed with histopathologically evaluation of sections taken from rat’s liver tissues.

According to histopathologic evaluations, when liver tissues of animals that is in control, 150 and 300 mg/kg FASB groups, which are two groups that only flower extract was administered, it was observed that a normal histologic feature similar to that of the control group. Apparent and negative structural alterations in contrast with the control group were observed in the liver tissue of the group subject to NDMA. It was determined that these alterations were liver hyperaemic, sinusoidal dilatation, perivascular cell infiltration, fibrosis in the portal area, degeneration and necrosis in the hepatocytes. It has been reported for the first time in this study that methanol extract of FASB can improve NDMA-induced toxic effects on liver. FASB treatment restored normal histologic structure of the liver through extensive regeneration of hepatocytes.

It was concluded as a consequence of *in vitro* studies that FASB flower includes significant amounts of vitamins A, C and E and that the flower may be an important vitamin source. As a consequence of mineral analyses, it was determined that the plant is rather rich in terms of mineral amount. It was observed that methanol extract of the flower has high total phenol, flavonoid content and in line with these values, the antioxidant capacity of the flower is at a high level. It was concluded that the flower has a strong antioxidant and antiradical activity because it inhibited hydroxyl radical, which the most reactive species is known in biological systems.

An increase observed in the liver tissue GSH-Px, SOD and CAT enzyme activities to which FASB (300 mg/kg) was administered together with NDMA in comparison with groups to which NDMA was administered suggests that the FASB plant which we used can be effective in preventing damage that occur in the liver. When livers of rats in NDMA +150 mg/kg FASB-administered group were examined, it was determined that negative structural alterations observed in NDMA-treated group declined and that a small number of perivascular cell infiltration, hyperaemia, degeneration in the hepatocytes consisted in liver sections. It was determined in the NDMA +300 mg/kg FASB group that negative structural alterations in the NDMA group reduced quite apparently and it was also observed as a result of the examination of tissue sections that the degree of degeneration decreased in hepatocytes and liver hyperaemic. When these results were evaluated, it was determined that overdoses of FASB flower is more effective in scavenging liver damage caused by NDMA. The fact that histologic alterations were observed in the NDMA group and that the damage incurred in groups to which the flower was applied together with NDMA allowed us to conclude that FASB flower is effective in preventing damages which may occur on liver tissue as a consequence of exposure to NDMA.

In conclusion, the study clearly demonstrated that vitamins A, C and E contents, the amount of mineral, antiradical, and antioxidant activity of FASB flower are quite high. Therefore, it was suggested that the flower can be used in preventing and treatment of several diseases which may be caused by ROS and that especially overdose 300 mg/kg of FASB can be used in preventing and treatment of liver diseases resulting from ROS. We suppose that FASB flower can be used as a protector antioxidant in foods due to its high vitamin A, C and E contents and high amounts of total phenol and flavonoid. We also think that our study will shed light on future studies that will be carried out.
